# Breaking the Code: Considerations for Effectively Disseminating Mass Notifications in Healthcare Settings

**DOI:** 10.3390/ijerph191811802

**Published:** 2022-09-19

**Authors:** Curt Harris, James Zerylnick, Kelli McCarthy, Curtis Fease, Morgan Taylor

**Affiliations:** Institute for Disaster Management, College of Public Health, University of Georgia, Athens, GA 30602, USA

**Keywords:** emergency communication, mass notification, overhead alerts, color codes, plain language, hospital, healthcare, emergencies, disasters

## Abstract

Many healthcare facilities use code-based alert systems to notify staff of ongoing emergencies via public announcement systems. This study sought to assess the ability of clinical and non-clinical employees across the State of Georgia to correctly identify their facility’s emergency codes, assess employee’s opinions of emergency alert systems, and identify significant predictors of emergency code identification accuracy. Anonymous electronic surveys asked 304 employees at five facilities to identify the codes for 14 different emergencies. Participants correctly identified the emergency codes with 44.37% accuracy on average. The codes for fire, infant abduction, and cardiac arrest were most commonly identified correctly. Code identification accuracy was significantly associated with training at orientation, knowledge of emergency code activation procedures, facility experience, and the total number of facilities in an employee’s career. Most survey participants favored a code-based alert system over a plain language-based alert system, citing concerns of causing panic in patients and visitors, and of maintaining confidentiality and discretion. The low code identification accuracy suggests healthcare employees may have limited awareness of ongoing emergencies. Transitioning to plain language overhead emergency alerts will better position employees, as well as patients and visitors, to effectively respond to emergencies and disasters occurring within a healthcare facility.

## 1. Introduction

Effective communication remains a significant challenge faced by healthcare facilities confronted with emergencies and disasters, regardless of whether it is a patient-specific emergency (such as a cardiac arrest) or a facility-wide emergency (such as a severe weather incident). When such an event occurs, facility leaders and emergency managers must coordinate communications internally among responding personnel and externally to patients, family members, visitors, and other unaffiliated individuals inside the facility. Initial communications to both groups most commonly occur in emergency alerts or warning systems.

An effective emergency alert must accomplish four goals: (1) reach the target audience; (2) capture the attention of the target audience; (3) ensure comprehension of the risk and necessary actions; and (4) lead the audience to respond appropriately [1]. Within the context of hospital emergencies, the target audience may only include a subset of personnel and visitors, or it may include everyone in the building. With few exceptions, these audiences do not typically search for and seek out warnings. As such, the alerts must be suitably conspicuous and possess qualities that encourage individuals to encode (e.g., read/hear, understand, personalize, store in memory) the content [2]. Warning systems commonly achieve this goal using a brief message accompanied by color, a signal word, and/or graphics [2]. Only after the message has been encoded can the target audience be sufficiently motivated to take the advised actions [1]. Lastly, an effective emergency alert system must distribute truthful notifications delivered by credible messengers [3,4]. High levels of trust have been repeatedly correlated with a reduction in uncertainty as well as an influence on risk perception and acceptance of risk [5]. Low-trust environments have also been correlated with a reduction in appropriate actions taken by the target audience [6]. Therefore, healthcare facility leaders and emergency managers must ensure their emergency alert systems issue accurate warnings that fully capture the attention of their target audiences and motivate those individuals to take the pertinent actions.

There is no national standard in the United States (U.S.) for such warning systems in healthcare facilities [7]. The Centers for Medicare & Medicaid Services (CMS) emergency preparedness regulations include a requirement for the facility to be able to coordinate patient care “within the facility, across healthcare providers, and with state and local public health departments and emergency management agencies and systems” [8]. However, there is no specific requirement for how such communication for non-patients should occur, nor does it explicitly call for hospitals to use a specific emergency alert system. Similarly, the 2022 Hospital Accreditation Standards from the Joint Commission (TJC) state that each facility must possess a communications plan that accounts “for the rapid evolution of an emergency or disaster and the need to provide consistently clear information regarding the emergency and the hospital’s ability to provide services both internally and externally” [9]. Training materials from TJC state that each healthcare organization must have a warning system “to communicate to employees about situations that may affect the safety of patients, visitors, and other employees” but do not specify a specific emergency alert system [10]. In fact, the publicly available resources for hospitals from TJC include guidance on multiple warning systems [11]. As a result of the lack of specific requirements, healthcare facility leaders often independently choose an emergency alert system that they believe will allow them to best maintain a safe environment of care [7].

One of the most popular emergency alert systems in a healthcare setting is the emergency code system, which refers to a series of code words used by hospital staff to quickly and discretely communicate an emergency and mobilize expert assistance [12,13,14]. The codes are designed to send specific and appropriate resources to the affected patient or incident victims [15]. Proponents of the code system argue that the codes are necessary to allow the facility to maintain the locus of control and to appropriately coordinate a response without informing patients, visitors, and other unaffected individuals in the facility [15,16].

Most code systems use color-based codes (e.g., “Code Red” for a fire), predicated on the idea that colors are easier to remember and serve as a tool to increase encoding among a target audience [2,14]. However, due to factors such as age, education, culture, and geographic area, individuals often associate specific colors with specific images that may not be universal [17,18,19]. This likely explains why colors that have less culturally engrained meanings, such as black, silver, purple, or orange, are used for a broader range of emergencies, while colors such as red and pink are nearly universally used for fire and pediatric incidents [14,16,20]. In fact, surveys of hospital emergency codes in California [12], Colorado [21], Florida [22], Oregon [13], and Puerto Rico [23] have indicated there is significant variation among different hospital emergency code systems. For example, the Colorado Hospital Association found 13 different codes among 59 reporting hospitals for bomb threats [21]. The different codes can cause confusion and secondary incidents, particularly in large-scale events involving multiple facilities and jurisdictions [7,12,24,25]. Staffing mechanisms at healthcare facilities further add to this confusion for emergency code systems. Approximately one in five healthcare workers has at least two jobs, and each employer may have its own emergency code designations [13,26,27].

Conversely, healthcare facilities also often use temporary employees to supplement clinical staffing. A 2021 survey from AMN Healthcare [28] reported that 95% of all surveyed healthcare facilities used temporary physicians, nurse practitioners, and/or physician assistants to address staffing shortages in the last 12 months. Even before the COVID-19 pandemic, 30% of total nursing hours across the U.S. were represented by supplemental clinical staffing [29]. It is easy for temporary employees, who have worked at multiple facilities with different code systems, to become confused and respond incorrectly to an announced code in an emergency [13]. Even without the high stress of emergencies, it is easy to become confused or spread misinformation [12,13]. In fact, healthcare employees consider the lack of consistency in codes to be a barrier to providing high-quality patient care [24].

Perhaps the most popular reasoning for a color-coded emergency alert system is the belief that it minimizes fear and panic among those not directly involved in responding to the incident [15]. A code-based system allows hospital staff to be separated from patients, visitors, and first responders during emergencies by providing each group with a different level of information regarding the threat [7]. However, extensive research into panic and anxiety in emergency communications suggests that messages that leave groups of the population without information during a crisis build fear and anxiety, regardless of the situation [30,31]. For example, “Code Blue” may indicate a patient requires resuscitation without notifying the patient’s family that their loved one needs such interventions. Healthcare providers historically have expressed discomfort regarding family proximity during resuscitation, citing concerns about a decreased quality of resuscitation and psychological traumatization of the family [32,33,34]. However, numerous studies have shown improved psychological outcomes for family members during witnessed resuscitation without any adverse effect on patient outcomes [35,36,37]. This example illustrates a foundational principle of emergency notifications: effective risk communication can mitigate negative individual behaviors in the population while simultaneously decreasing anxiety [38].

To minimize these concerns, hospital associations from 25 of the 50 states in the U.S. have recommended the introduction of a standardized set of emergency codes [39]. There is little question that standardized codes would result in reduced training time, misunderstandings, and a more efficient overall response [20]. However, little progress has been made in implementing these recommendations, largely due to a lack of consensus. Of the 25 states, 12 have advocated for plain language codes in hospitals, while the remaining 13 remain focused on standardized codes [7]. There is little agreement among those recommending a standardized set of emergency codes. For example, the California Hospital Association’s system has 11 color codes, which some healthcare professionals feel is excessive [12,20]. Some recommendations explicitly state facilities should not use codes similar to types used by other organizations, such as Code Amber for a missing child, while other systems use Code Amber for missing child [13,20]. Yet others have argued that the three basic codes of red (for fire), blue (for medical emergency), and pink (for infant abduction) should be supplemented by individual facility codes [20]. Even the recommendations published by TJC do not align with any of the previously discussed systems [11]. As the debate continues, applying emergency codes only in color, or color-based emergency codes including some plain language, are still common practices worldwide [14].

Few studies, both nationally and internationally, have examined the ability of hospital employees to accurately identify their facility’s emergency color codes. In a study focused on the Delaware Valley region, Mapp et al. [24] reported 77% accuracy among study participants, which only included clinical providers. In South Korea, participating nurses in one hospital identified emergency color codes with 87.4% average accuracy [40], and participating clinical providers across four general hospitals averaged 59.4% accuracy [14]. As of July 2022, no study has examined the accuracy of employees’ knowledge of emergency codes in the State of Georgia.

To address this gap in the literature, this study aimed to assess the efficacy of color code emergency alert systems in hospitals in the State of Georgia. At the time of the study, numerous hospitals across the state used different notification systems and/or colors within a code-based notification system. Therefore, the main objective was to determine the ability of clinical and non-clinical employees to identify their facility’s emergency codes correctly. Secondary objectives included assessing employees’ opinions of emergency alert systems and identifying characteristics that are significant predictors of emergency code identification accuracy. It was hypothesized that code identification accuracy would be poor across clinical and non-clinical employees, with experience in healthcare and the total number of facilities in a career being significant predictors of a worse accuracy score. It was also hypothesized that employees would still prefer a code-based emergency notification system instead of a plain language system.

## 2. Materials and Methods

### 2.1. Sampling and Data Collection

This quantitative study assessed clinical and non-clinical staff knowledge of emergency codes in five healthcare facilities across the State of Georgia. The healthcare facilities were specifically chosen to represent hospital size, capacity, and communities served among healthcare facilities in the State of Georgia, using non-plain language codes to communicate emergency information to staff. These facilities included two level I trauma facilities (Facilities A and D), one level II trauma facility (Facility C), and two acute care facilities (Facilities B and E), one of which has an attached nursing home (Facility B). The study comprised facilities in rural and urban geography; small, medium, and large bed capacities and staffing levels; and variance of services offered. The emergency coordinator at each participating facility received approval from central administration prior to conducting the study.

On the day of the study, researchers arrived at the healthcare facility unannounced to all staff except for the emergency coordinator and central administration. The researchers worked with the emergency coordinator to develop a plan to survey each department and service within the facility over a single day, using a convenience sampling strategy. This process typically lasted about four to six hours for each facility. This strategy primarily focused on route efficiency planning and identifying staff locations within each department and service. Because involvement in the study was voluntary, the sample size was determined by the number of eligible employees that chose to participate. The survey was conducted via an electronic format. Researchers utilized laptops and tablets to complete the survey due to their portable nature and ease of use. Participants were asked to remove their badges and place them in their pockets to ascertain code knowledge from memory only, without using quick-reference guides (often printed on the back of hospital identification badges). Inclusion criteria for this study were: being 18 years of age or older; and being an active employee at the facility. The only exclusion criterion were central administration personnel due to their prior knowledge of the survey.

### 2.2. Survey Design

The survey was developed using the web-based surveying tool, Qualtrics. It included three parts: demographic information, code identification, and experience with hospital emergency codes, which are described in further detail below. The complete survey is available in the supplemental materials.

#### 2.2.1. Demographic Information

The first part of the survey collected only work-related demographic data to protect the anonymity of all participants. Collected demographics include clinical or non-clinical role, typical shift, years employed at the current facility, years of experience in healthcare, and the total number of facilities in participant’s career. The participants also scored their confidence in their knowledge of the emergency codes at their facility on a Likert scale from one to seven, with one being “not confident at all” and seven being “very confident”.

#### 2.2.2. Code Identification

The second part of the survey asked participants to identify the code for 14 different emergencies, including fire, cardiac arrest, pediatric emergency, bomb threat, violence, active shooter, hostage situation, infant abduction, mass casualty, internal disaster, hazardous materials, missing adult, missing child, and severe weather. These emergencies are a comprehensive list for which a code was specified in the emergency operations plan for at least one of the participating facilities. For each emergency, the participant was first asked if the code type was color code, plain language, other (e.g., Code John, Code Triage), or none identified. The participant was also able to indicate if they were unsure. If the participant indicated the code type was a color code, they were then given a list of 20 colors from which to choose the appropriate color code. If the participant indicated the code type was plain language or other (e.g., Code John, Code Triage), they were then provided with a free-response box to write the code. If the participant indicated there was no code identified or if they were unsure of the code type, the survey moved to the next emergency.

#### 2.2.3. Experience with Hospital Emergency Codes

The final part of the survey asked the participants about their experience with hospital emergency codes. This included questions on knowledge of procedures to activate a code, preference for plain language or color codes, frequency of training on current codes, if they have worked at other healthcare facilities with different codes, and if they have witnessed code confusion during drills and/or real-world emergencies. The participants also reevaluated their confidence in their knowledge of the emergency codes at their facility, using the same Likert scale as the pre-survey confidence scale.

### 2.3. Ethical Considerations

This study was approved by the University of Georgia’s Institutional Review Board (IRB) (Protocol No. 00005234). Written, informed consent was obtained at the beginning of the survey. Participation in the study was voluntary, and the consent form clearly stated that if an employee chose not to participate or stopped in the middle of the survey, it would have no bearing on their employment or performance evaluation. No identifying information about the participants was obtained, and the survey results remained confidential throughout data collection and analysis.

### 2.4. Data Analysis

Each facility’s emergency plan was consulted to identify the accurate codes for each emergency in the survey, and participants’ responses were assessed for accuracy in their code identification. Frequencies of responses are presented as percentages, and significance levels were set to 0.05. For each participant, the code identification score is expressed as the percent of emergencies where the code was correctly identified. It was then treated as a typical grade, with possible values ranging from 0 to 100. A paired t-test was performed to assess the significance of the change in confidence scores pre-survey and post-survey. Cohen’s d was calculated to estimate the effect size of the change in confidence scores. Using both code identification score and change in confidence score as outcomes, one-way analysis of variance (ANOVA) tests were conducted to determine the significance of mean differences between groups in the demographic characteristic and experience with code variables. The change in the confidence score outcome was calculated as the difference between the post-survey self-assessed confidence score and the pre-survey self-assessed confidence score. All groups were evaluated for normality of residuals and homoskedasticity. Tukey’s Honestly Significant Difference post hoc test was conducted for variables that had a significant difference in the group means. A univariate linear regression analysis was conducted with all covariates to identify variables significantly associated with code identification score. The significantly associated covariates were then included in a multivariate regression analysis to identify significant predictors of code identification score.

### 2.5. Software

All analyses were performed using R Statistical Software (v4.2.1) (R Foundation for Statistical Computing, Vienna, Austria) [41], via Rstudio Desktop (RStudio, Inc., Boston, MA, USA) [42] on a Windows Server operating system (Microsoft Corporation, Redmond, WA, USA) [43]. The following R packages were utilized: agricolae [44], broom [45], effsize [46], ggpattern [47], extrafont [48], gtsummary [49], here [50], readxl [51], rstatix [52], scales [53], table1 [54], tidyverse [55], and writexl [56]. All code developed for this study, including specific use of each package, can be found in the supplemental materials.

## 3. Results

### 3.1. Sample Characteristics

A total of 304 employees participated in the study (Table 1). With reference to facilities, employees of the level I trauma centers (Facilities A and D) accounted for 58% of the participants, followed by employees of the acute care facilities, (Facilities B and E, 22%), and the level II trauma center, (Facility C, 20%). Almost two-thirds (64%) of the participants were clinical staff, and the vast majority (92%) primarily worked day shifts. The participants were relatively evenly distributed across years of experience at their current facility, and nearly half (49%) of the participants had worked in healthcare for more than eight years. Three-quarters (75%) of the participants have worked at three or fewer facilities in their careers.

When asked about their experience with emergency codes, approximately two-thirds (69%) of the study participants indicated familiarity with the procedures associated with activating emergency codes (Table 2). Similar proportions did not report witnessing any confusion about emergency code meanings during their duties (64%) or having worked at a different facility with different codes (65%). More than two-thirds (68%) of participants received training on emergency codes at orientation, and more than half (56%) reported code training through drills and exercises. Approximately half (51%) of the participants reported receiving annual training on emergency codes, and less than a quarter (19%) of participants reported more than a year since their last training for emergency codes.

Nearly two-thirds (65%) of participants indicated a preference for maintaining color codes in their facility (Table 2). The most common justifications for keeping color codes included preventing panic in patients/visitors (51 participants, 17%), and maintaining confidentiality and discretion (24 participants, 8%). In contrast, among the 35% of participants that preferred switching to plain language codes, the most common reasonings included that plain language codes require no memorization (57 participants, 19%), and allow everyone in the facility, including patients and visitors, to know what the code means (21 participants, 7%). More than three-quarters (79%) of participants stated that certain emergencies should always have a color code. Among these participants, 89 (46%) responses stated that cardiac arrest should remain an exception, followed by 77 (40%) for infant abduction, 73 (38%) for fire, and 63 (33%) for active shooter. Six (3%) participants indicated that all codes should be transitioned to color codes, including ones that are not currently codes, and another 12 (6%) participants identified color codes that should remain exceptions but did not exist at their facility.

### 3.2. Facility Codes

Among the participating facilities, the emergency code meanings have little consistency (Table 3). Cardiac arrest, fire, hazardous materials, and infant abduction have the same color code across all five facilities. However, “Code Pink” could refer to infant abduction or missing child for four of the participating facilities. Similarly, depending on the facility, “Code Silver” could indicate active shooter or hostage situation, while “Code Gray” could indicate bomb threat, hostage situation, missing adult, severe weather or violence. These inconsistencies are not limited to color codes, either. Depending on the facility, the code for a mass casualty incident could be either “Code Triage” or “Code Triage A”, while the code for an internal disaster could be “Code Triage”, “Code Triage B”, or “Code [Facility Name].” None of the participating facilities had an emergency code for a pediatric emergency, and two of the five facilities did not have one for a bomb threat. Plain language is only used for severe weather by two of the participating facilities.

### 3.3. Code Identification Accuracy

**Figure 1:**
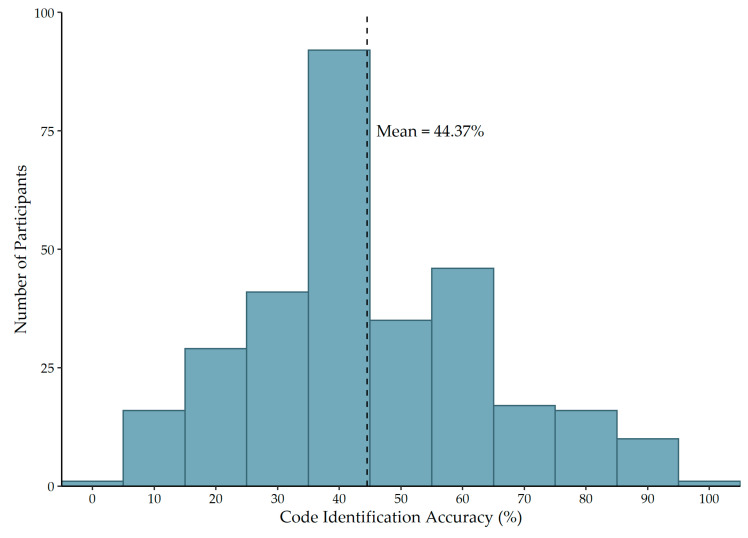
Distribution of emergency code identification accuracy score.

**Figure 2:**
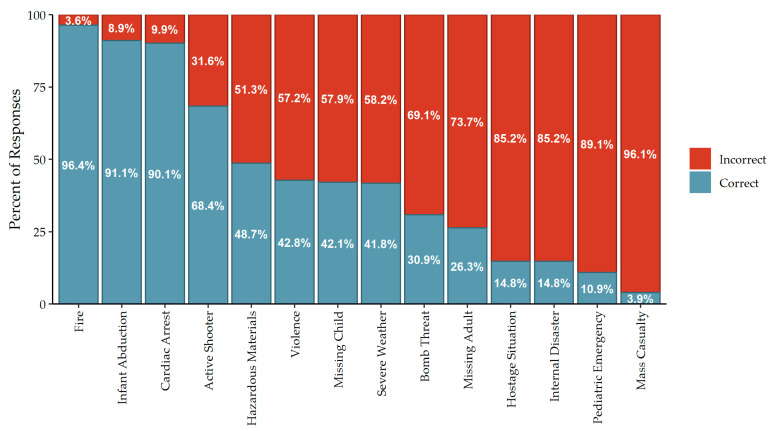
Code identification accuracy by emergency across all participants.

Figure 1 illustrates the distribution of code identification accuracy per participant, calculated as the proportion of emergencies for which the participant correctly identified their facility’s emergency code. The average accuracy score across all participants was 44.37% (SD = 19.11%). Figure 2 summarizes the accuracy of code identification across the 14 emergencies included in this study. The codes corresponding to fire, infant abduction, and cardiac arrest were correctly identified by more than 90% of participants. The codes corresponding to hostage situation, internal disaster, pediatric emergency, and mass casualty incident were incorrectly identified by more than 85% of participants.

### 3.4. Code Identification Score Associations

Table 4 includes the results of the one-way ANOVA analyses for the demographic characteristic variables with code identification score. The facility effect was significant (F = 33.28; d.f. = 4, 299; *p* < 0.001). Pairwise comparison of the means using Tukey’s Honestly Significant Difference procedure indicated the average code identification score for participants from Facility B were significantly higher than for participants from Facilities A, D, and E (F = 36.23; d.f. = 4, 299; *p* < 0.001). There was no significant difference in the average code identification score for participants from Facilities B and C. The post hoc analysis also indicated that the average code identification score for participants from Facility D were significantly higher than for participants from Facilities A and E (F = 36.23; d.f. = 4, 299; *p* = 0.001). There was no significant difference in the average code identification score for participants from Facilities A and E.

Years of experience in healthcare also had a significant effect on code identification score (F = 3.80; d.f. = 3, 300; p = 0.010). Post hoc analyses using the Tukey post hoc criterion for significance indicated that the average code identification score for participants with five to eight years of experience in healthcare were significantly higher than for participants with zero to two years of experience and for participants with two to five years of experience (F = 3.65; d.f. = 3, 300; p = 0.025). There was no significant difference in the average code identification score for participants with more than eight years of healthcare experience compared to any other group.

The average code identification score of the four categories of experience at the current facility were unequal according to the one-way ANOVA (F = 3.00; d.f. = 3, 300; *p* = 0.031). Pairwise comparison of the means using Tukey’s Honestly Significant Difference procedure indicated one marginally significant comparison: participants with more than eight years of experience (M = 47.14%) scored 6.98 percentage points higher on average (95% CI = −0.09%, 14.06%; *p* = 0.054) than participants with less than two years of experience at the current facility (M = 40.16%). The other comparisons were not significant. Code identification scores did not differ significantly with position type, typical shift, or total facilities in the career.

Table 5 summarizes the results of the one-way ANOVA analyses for the experience with emergency codes variables with code identification score. Participants who knew their facility’s emergency code activation procedures (M = 48.5%, SD = 19.8%) scored significantly higher (F = 31.76; d.f. = 1, 302; *p* < 0.001) than those who did not know their facility’s activation procedures (M = 35.6%, SD = 14.6%). Participants who preferred color codes (M = 46.2%, SD = 19.4%) scored significantly higher (F = 4.70; d.f. = 1, 302; *p* = 0.031) than those who preferred plain language codes (M = 41.2%, SD = 18.6%). Similarly, participants who advocated for color code exceptions under a plain language system (M = 46.9%, SD = 19.1%) scored significantly higher (F = 18.3; d.f. = 1, 302; *p* < 0.001) than those who did not believe there should be any exceptions under a plain language system (M = 35.6%; SD = 17.1%).

The 197 participants that did not report working at a different facility with different codes had an average score of 46.3% (SD = 19.2%). The 80 participants who reported working at a different facility with different codes had an average score of 41.2% (SD = 19.5%). The 27 participants, unsure if they had worked at a different facility with different codes, had an average score of 41.0% (SD = 17.6%). The effect of working at a different facility with different codes, therefore, was only marginally significant (F = 2.57; d.f. = 2, 301; *p* = 0.078). Code identification scores did not differ significantly depending on whether the participant had witnessed code confusion.

Training on emergency codes had a significant effect on code identification scores. Participants who received training at orientation (M = 46.3%, SD = 19.7%) scored significantly higher (F = 5.78; d.f. = 1, 302; *p* = 0.017) than those who did not receive training at orientation (M = 40.6%, SD = 17.7%). Participants who reported code training during emergency drills and/or exercises (M = 46.5%, SD = 19.0%) scored significantly higher (F = 4.11; d.f. = 1, 302; *p* = 0.044) than those who reported no code training during emergency drills and/or exercises (M = 42.0%; SD = 19.4%). Participants who reported annual training for codes (M = 49.0%, SD = 20.1%) scored significantly higher (F = 18.12; d.f. = 1, 302; *p* < 0.001) than those who did not report annual training (M = 39.8%, SD = 17.2%). The difference of means for time since last training on codes was marginally significant (F = 2.16; d.f. = 3, 300; *p* = 0.093).

Table 6 summarizes the results from the univariate and multivariate analyses with code identification score. The univariate analysis showed that facility, shift type, facility experience, healthcare experience, number of facilities in career, knowledge of a facility’s code activation procedures, a history of working at a different facility with different codes, code type preference, code exceptions for plain language, code training at orientation, code training during drills and/or exercises, and annual code training were all independently significantly associated with code identification score.

As indicated in Table 6, the multivariate regression model showed that facility, facility experience, number of facilities in career, knowledge of code activation procedures, and code training at employee orientation were significant predictors of code identification score when adjusting for all other significant covariates. Compared to Facility A, employees of Facility B scored approximately 25.6 percentage points higher (95% CI: 18.3, 33.0; *p* < 0.001) when adjusting for all other covariates. Similarly, employees from Facilities C and D scored 21.7 percentage points (95% CI: 16.4, 27.0; *p* < 0.001) and 15.9 percentage points (95% CI: 11.1, 20.8; *p* < 0.001), respectively higher than employees from Facility A when holding all of the remaining covariates constant. There was no significant difference in code identification scores between Facilities A and E employees. These differences do not correspond to facility size, location (i.e., urban vs. rural), or trauma designation.

Employees who had worked at their current facility for two to five years scored approximately 5.85 percentage points higher (95% CI: 0.028, 11.7; *p* = 0.05) than employees who had worked at their current facility for less than two years when adjusting for all other covariates. However, there was no significant difference in code identification scores for employees who worked at their current facility for more than five years compared to employees who worked at their current facility for less than two years when adjusting for all other covariates.

Study participants who had worked at four or five healthcare facilities in their careers scored approximately 6.72 percentage points lower (95% CI: 0.579, 12.9; *p* = 0.033) than participants who had only worked at one facility in their careers, holding the all other variables constant. Similarly, employees working at more than five facilities in their careers scored marginally significantly lower (*p* = 0.067) than employees who had only worked at one facility in their careers when holding all of the remaining variables fixed. When adjusting for all other covariates, there was no significant difference between working at one facility and two to three facilities in the study participants’ careers.

Participants who reported knowledge of code activation procedures scored approximately 9.07 percentage points higher (95% CI: 5.01, 13.1; *p* < 0.001) than participants who did not, holding all other covariates constant. Participants who received training on codes at employee orientation scored approximately 4.2 percentage points higher (95% CI: 0.316, 8.09; *p* = 0.035) than participants who did not receive training at orientation when holding all other covariates constant. There was no significant difference in code identification scores for other types of training after adjusting for all remaining covariates.

### 3.5. Confidence Scores

**Figure 3:**
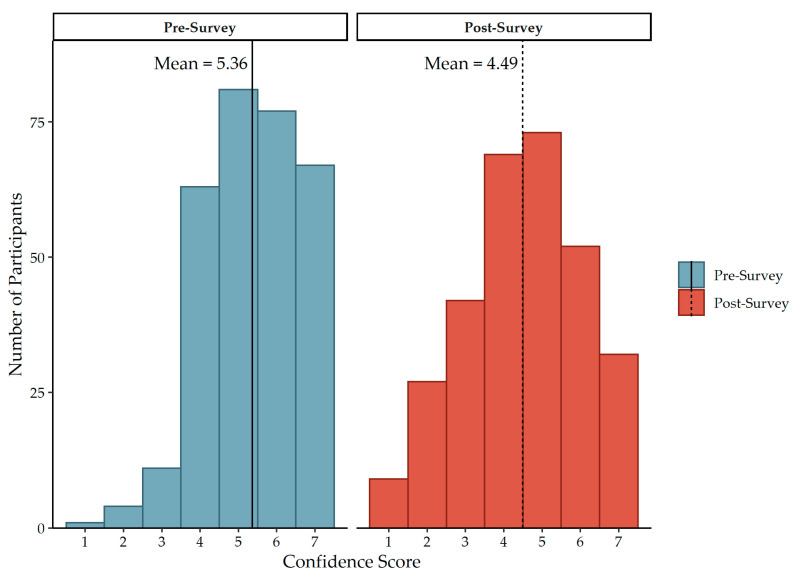
Distribution of confidence score, before and after code identification survey.

**Figure 4:**
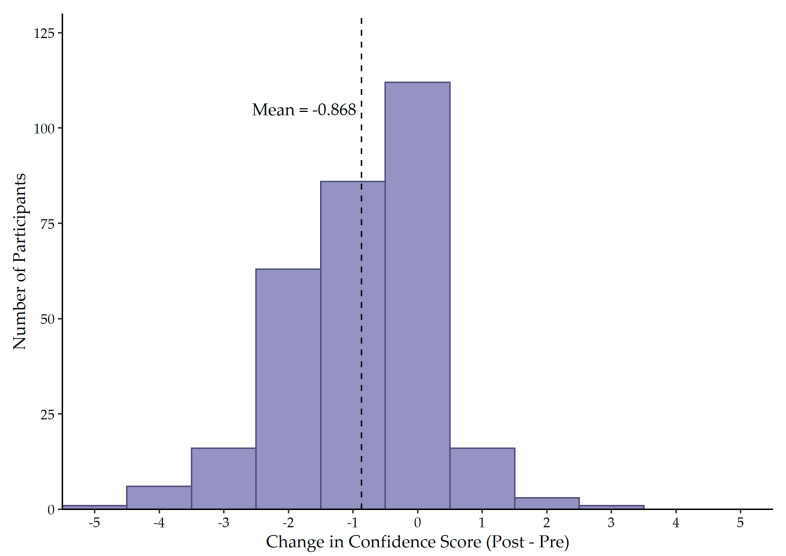
Distribution of change in confidence score.

Prior to completing the survey, participants rated their confidence in their knowledge of the emergency codes at their facility as 5.36 on average (SD = 1.24), on a Likert scale from one to seven, with one being “not confident at all” and seven being “very confident” (Figure 3). Following survey completion, participants rated their confidence as 4.49 on average (SD = 1.55) (Figure 3). Completing the survey resulted in a decrease in confidence of code knowledge (t = 13.005; df = 303, *p* < 0.001) by 0.868 points (95% CI: 0.737, 0.999) (Figure 4). The average confidence score post-survey is 0.605 standard deviations (95% CI: −0.704, −0.505) below the average confidence score pre-survey.

### 3.6. Change in Confidence Score Associations

Table 4 includes the results of the ANOVA analyses for the demographic characteristic variables with the secondary outcome, change in confidence score, which calculated the difference between each participant’s post-survey confidence score and the pre-survey confidence score. The facility effect was marginally significant (F = 2.00, d.f. = 4, 299; *p* = 0.095). Change in confidence score did not differ significantly with position type, typical shift, facility experience, healthcare experience, or total facilities in career.

Table 5 includes the results of the ANOVA analyses for the experience with emergency codes variables with a change in confidence score. Participants who reported annual training for codes (M = −0.73, SD = 1.08) had a significantly smaller decrease in confidence score (F = 4.59; d.f. = 1, 302; *p* = 0.033) than those who did not report annual training (M = −1.01, SD = 1.23). The change in confidence scores did not differ significantly with knowledge of code procedures, witnessing code confusion, working at a different facility with different codes, code type preference, color code exceptions in a plain language system, code training at orientation, code training during drills and/or exercises, or time since last code training.

## 4. Discussion

This study showed that both clinical and non-clinical employees have limited accuracy in identifying their hospital’s emergency codes. Code identification accuracy was significantly associated with training at orientation, knowledge of emergency code activation procedures, facility experience, and total facilities in the career. The majority of survey participants favored a code-based alert system over a plain language-based alert system, citing concerns of causing panic in patients and visitors and maintaining confidentiality and discretion.

The emergency codes assigned to ten of the fourteen emergencies in this study varied across the five participating facilities. Only codes for cardiac arrest (Code Blue), fire (Code Red), hazardous materials (Code Orange), and infant abduction (Code Pink) were consistent. These four codes mostly reflect the code recommendations by TJC, except Code Orange, which TJC specifies is only for medical decontamination [11]. Additionally, codes for active shooter, bomb threat, hostage situation, and violence were all slightly different across facilities. Given the similar nature of such incidents, employees working at multiple facilities would be more likely to confuse Code Black, Code Gray, and Code Silver, let alone other codes such as “Security Stat” and “Code Strong.” These variations in codes are reflected extensively in other studies [12,13,14,16,21,22,23,40]. Code Blue for cardiac arrest or medical emergency is largely consistent across these publications, except that Puerto Rico uses Code Green [23]. While most facilities in the U.S. use Code Red for fire, Code Red is also used for pediatric cardiac arrest in one facility in South Korea [14] and internal disaster in 25% of participating facilities in a Saudi Arabian study [16]. No participating facility had a code for pediatric emergency, contrary to standards published in other states [12,13,21]. However, the recommendations from TJC do not include pediatric emergency [11].

Study participants correctly identified their facility’s emergency codes with 44.37% accuracy on average, which is significantly lower than in other existing publications [14,24,40]. Few studies have assessed employee knowledge of healthcare facility emergency codes, particularly within the U.S.. In a study focused on the Delaware Valley region, Mapp et al. [24] reported 77% accuracy among study participants. In South Korea, participants in one hospital identified emergency color codes with 87.4% average accuracy [40], and participants across four general hospitals averaged 59.4% accuracy [14]. This discrepancy may partially be explained by the difference in the number of codes at the participating facilities. In the five Georgia facilities included in this study, either twelve or thirteen emergency codes were used. In contrast, the facility in Jeong and Lee [40] used eight codes, and the facilities in Lee and Lee [14] used between two and nine emergency codes. There were also more participants in the present study, which may further contribute to the difference from existing studies.

Codes for fire, infant abduction, and cardiac arrest were most commonly identified correctly among study participants, which reflects the results of Lee and Lee [14] and Jeong and Lee [40]. Nearly half of the study participants (48.7%) correctly identified “Code Orange” as the emergency code for a hazardous materials incident, which was higher than the 38.3% of participants in Lee and Lee [14] but less than the 88.9% of participants in Jeong and Lee [40]. Even though all participating facilities used the same code for a hazardous materials incident, more participants correctly identified the code for an active shooter. It is plausible this increase is due to the fact that facilities train more frequently on active shooter incidents or that the code for an active shooter is the newest code due to current events [57]. Alternatively, employees may be more likely to encode the alert for an active shooter due to the fear of interpersonal violence [58,59]. Participants were less aware of codes for situations such as missing adults (26.3%), hostage situations (14.8%), internal disasters (14.8%), and mass casualty incidents (3.9%). Similar levels of awareness for mass casualty were reported in Lee and Lee [14], but Jeong and Lee [40] reported 84.2% accuracy for “in and out of hospital disaster.” The rare nature of such incidents likely contributes to the lack of awareness of these codes, which suggests that a combination of training and conversion to a plain language system may be necessary for a timely and effective response [7,13,16,20,24].

Code identification accuracy was significantly higher in participants who received training at employee orientation, had knowledge of emergency code activation procedures, and had worked at their current facility for two to five years. However, accuracy was lower in employees who had worked at four to five facilities in their careers, suggesting that code confusion becomes particularly pronounced after having more than three healthcare employers. This trend may also explain why fewer participants reported witnessing code confusion or working at a facility with different color codes, as less than a quarter of study participants reported working at more than three facilities in their careers. While few other studies have examined the relationship of employee characteristics with emergency code knowledge, Jeong and Lee [40] found that code accuracy was significantly higher in nurses aged 40 years old or older, who had worked in healthcare for at least 10 years, and who had experience in disaster response. As this study did not find a significant relationship between years of experience in healthcare and code identification accuracy, further studies should be conducted to better understand the characteristics of employees with an increased knowledge of emergency code meanings.

Participants’ confidence in their knowledge of the emergency codes at their facility decreased by an average of 0.868 points (95% CI: 0.737, 0.999) following the completion of the survey, with the post-survey confidence score averaging 4.49 (SD = 1.55) out of seven. This post-survey confidence score is similar to Jeong and Lee [40], whose participants reported an average confidence score of 3.30 out of five. Among this study’s participants, those who reported annual training on emergency codes had a significantly smaller decrease in confidence scores than those who did not report annual training. This difference further emphasizes the need for effective, recurring training, as increasing employee’s confidence in their knowledge of emergency codes will significantly improve the efficiency of their response to emergency situations [40,60,61].

Another method to increase employee’s confidence in and accuracy of emergency codes is to transition to a plain language alert system. While there are no national standards or requirements for healthcare facilities, both the U.S. Federal Emergency Management Agency (FEMA) and the Department of Health and Human Services (DHHS) advocate for plain language communications in all emergency and disaster communications [7,31]. The Hospital Incident Command System (HICS) reflects such an emphasis, but overhead emergency alerts within a facility have yet to be included in this philosophy [7,31]. This disconnect may plausibly be explained by the healthcare sector remaining fairly separated from FEMA. Thus, FEMA’s unequivocal recommendations in favor of plain language communications may not be reaching healthcare employees. The hesitancy expressed by this study’s participants to switch to such a system reflects those that have been well documented in other publications [7,14,20,24,31]. In particular, participants in the study cited avoiding panic in patients and visitors as well as maintaining confidentiality and discretion as the primary reasons to retain a code-based emergency alert system. However, numerous studies have demonstrated that a lack of clear communication further increases panic during emergencies that would affect the safety of patients and visitors in a healthcare facility [7,20,31,38]. Further, plain language alerts, particularly those regarding medical emergencies for specific patients, do not violate the Health Insurance Portability and Accountability Act (HIPAA), as these alerts do not include any of the 18 identifiers [62]. HIPAA even allows for identifiers to be included in communications in the case of an emergency [63]. Advocates of a code-based alert system in this study also stated that, even under a primarily plain language-based alert system, color codes should remain in place for cardiac arrest (Code Blue), infant abduction (Code Pink), and fire (Code Red). The reasoning for such exceptions was split among participants, with some stating they should remain for confidentiality purposes, while others said they should remain because they are already widely accepted and used. Mapp et al. [24] found the majority of patients knew the meaning of “Code Blue” and “Code Red.” Further, the color pink is most commonly associated with situations involving children, which suggests patients and visitors may be able to infer the meaning of “Code Pink” [14]. While the benefit of transitioning to a plain language overhead emergency alert system has been well-documented, this study suggests there remains a significant need for training and education to translate the results of such research into practice.

The generalizability of this study was limited as the data were collected in five Georgia hospitals using convenience sampling. Efforts were made to minimize sampling bias as much as possible during the planning meeting with each facility emergency manager to ensure each department was covered. However, the distribution of the clinical versus non-clinical personnel among study participants suggests this sampling strategy may not have adequately covered non-clinical employees. Recall bias may have affected participant responses, as employees who had memorable experiences with certain codes may have remembered the meaning more than others. Response bias may have also affected participant responses, as there were anecdotal reports of employees studying color codes before researchers arrived in their department as word spread throughout the facility on the day of the study. However, given the overall low accuracy scores, it is unlikely this bias significantly affected the results. In future studies, a larger sample size with participants from various states or regions should be conducted to increase the external validity of the study findings. Secondly, future studies could also further examine potential emergency code knowledge differences among healthcare providers (e.g., physicians, nurses, etc.) as well as among non-clinical. Thirdly, the unique efficacy of various training methods—such as virtual, in-person lectures, and in-person hands-on—should be further explored to better examine the influence of training on code identification performance. Fourthly, given the contrast between the published literature and healthcare providers’ opinions, the origins and reasonings for healthcare provider hesitancy for plain language alerts should be further investigated. Lastly, research should also be conducted into methods to effectively address misconceptions regarding emergency and plain language codes.

## 5. Conclusions

Transitioning to plain language overhead emergency alerts instead of color codes in healthcare settings will reduce confusion in employees and panic in visitors and patients, contrary to traditional beliefs by healthcare providers. This study demonstrated poor overall knowledge of emergency alert codes in five Georgia healthcare facilities, highlighting the need for readily accessible job aids and pocket guides. Of the fourteen emergencies included in the study, codes for fire, infant abduction, and cardiac arrest were most often identified correctly. Training and experience at the facility were significantly associated with higher identification accuracy scores, while a history of more than three healthcare employers was significantly associated with lower identification accuracy scores. The study participants also expressed apprehension about a plain language overhead emergency alert system, citing concerns regarding panic, fear, confidentiality, and discretion. As such, it is recommended that future research should focus on improving training and educational methods to increase healthcare employee support of plain language emergency alert systems. Transitioning to a plain language emergency alert system will better position healthcare employees, patients, and visitors to respond to emergencies and disasters effectively.

## Figures and Tables

**Table 1:** Survey participant demographic characteristics.

	Facility					
	**A**, N = 99 ^1^	**B**, N = 21 ^1^	**C**, N = 59 ^1^	**D**, N = 78 ^1^	**E**, N = 47 ^1^	**Overall**, N = 304 ^1^
**Position Type**						
Clinical	74 (75%)	14 (67%)	39 (66%)	49 (63%)	20 (43%)	196 (64%)
Non-clinical	25 (25%)	7 (33%)	20 (34%)	29 (37%)	27 (57%)	108 (36%)
**Typical Shift**						
Day Shift	88 (89%)	19 (90%)	56 (95%)	71 (91%)	45 (96%)	279 (92%)
Night Shift	1 (1.0%)	1 (4.8%)	0 (0%)	2 (2.6%)	1 (2.1%)	5 (1.6%)
Equal times on both shifts	10 (10%)	1 (4.8%)	3 (5.1%)	5 (6.4%)	1 (2.1%)	20 (6.6%)
**Facility Experience**						
0–2 years	28 (28%)	4 (19%)	14 (24%)	31 (40%)	13 (28%)	90 (30%)
2–5 years	30 (30%)	8 (38%)	12 (20%)	13 (17%)	14 (30%)	77 (25%)
5–8 years	8 (8.1%)	0 (0%)	8 (14%)	9 (12%)	7 (15%)	32 (11%)
>8 years	33 (33%)	9 (43%)	25 (42%)	25 (32%)	13 (28%)	105 (35%)
**Healthcare Experience**						
0–2 years	15 (15%)	1 (4.8%)	7 (12%)	21 (27%)	7 (15%)	51 (17%)
2–5 years	26 (26%)	5 (24%)	11 (19%)	11 (14%)	14 (30%)	67 (22%)
5–8 years	10 (10%)	2 (9.5%)	8 (14%)	9 (12%)	8 (17%)	37 (12%)
>8 years	48 (48%)	13 (62%)	33 (56%)	37 (47%)	18 (38%)	149 (49%)
**Total Facilities in Career**						
1 facility	31 (31%)	6 (29%)	34 (58%)	28 (36%)	24 (51%)	123 (40%)
2–3 facilities	44 (44%)	9 (43%)	16 (27%)	20 (26%)	17 (36%)	106 (35%)
4–5 facilities	10 (10%)	3 (14%)	5 (8.5%)	21 (27%)	3 (6.4%)	42 (14%)
>5 facilities	14 (14%)	3 (14%)	4 (6.8%)	9 (12%)	3 (6.4%)	33 (11%)

^1^ n (%).

**Table 2:** Survey participant experience with emergency codes.

	Facility					
	**A**, N = 99 ^1^	**B**, N = 21 ^1^	**C**, N = 59 ^1^	**D**, N = 78 ^1^	**E**, N = 47 ^1^	**Overall**, N = 304 ^1^
**Knowledge of Code Activation Procedures**						
No	32 (32%)	5 (24%)	14 (24%)	24 (31%)	19 (40%)	94 (31%)
Yes	67 (68%)	16 (76%)	45 (76%)	54 (69%)	28 (60%)	210 (69%)
**Witnessed Code Confusion**						
No	53 (54%)	12 (57%)	44 (75%)	50 (64%)	37 (79%)	196 (64%)
Yes	46 (46%)	9 (43%)	15 (25%)	28 (36%)	10 (21%)	108 (36%)
**Worked at a Facility with Different Color Codes**						
No	55 (56%)	14 (67%)	44 (75%)	50 (64%)	34 (72%)	197 (65%)
Yes	31 (31%)	5 (24%)	12 (20%)	22 (28%)	10 (21%)	80 (26%)
Maybe	13 (13%)	2 (9.5%)	3 (5.1%)	6 (7.7%)	3 (6.4%)	27 (8.9%)
**Code Type Preference**						
Color Codes	52 (53%)	16 (76%)	45 (76%)	58 (74%)	28 (60%)	199 (65%)
Plain Language	47 (47%)	5 (24%)	14 (24%)	20 (26%)	19 (40%)	105 (35%)
**Code Exceptions for Plain Language**						
No	35 (35%)	2 (9.5%)	5 (8.5%)	9 (12%)	13 (28%)	64 (21%)
Yes	64 (65%)	19 (90%)	54 (92%)	69 (88%)	34 (72%)	240 (79%)
**Training at Orientation**						
No	28 (28%)	8 (38%)	11 (19%)	29 (37%)	20 (43%)	96 (32%)
Yes	71 (72%)	13 (62%)	48 (81%)	49 (63%)	27 (57%)	208 (68%)
**Training During Drills/Exercises**						
No	42 (42%)	6 (29%)	29 (49%)	36 (46%)	22 (47%)	135 (44%)
Yes	57 (58%)	15 (71%)	30 (51%)	42 (54%)	25 (53%)	169 (56%)
**Annual Training**						
No	62 (63%)	7 (33%)	17 (29%)	38 (49%)	25 (53%)	149 (49%)
Yes	37 (37%)	14 (67%)	42 (71%)	40 (51%)	22 (47%)	155 (51%)
**Time Since Last Training**						
<1 month	23 (23%)	4 (19%)	6 (10%)	11 (14%)	7 (15%)	51 (17%)
1–6 months	21 (21%)	11 (52%)	27 (46%)	34 (44%)	20 (43%)	113 (37%)
6–12 months	25 (25%)	5 (24%)	18 (31%)	20 (26%)	15 (32%)	83 (27%)
>1 year	30 (30%)	1 (4.8%)	8 (14%)	13 (17%)	5 (11%)	57 (19%)

^1^ n (%).

**Table 3:** Codes for each emergency type, as identified by each facility’s emergency operations plan.

	Facility				
	A	B	C	D	E
**Active Shooter**	Color Code **Black**	Color Code **Silver**	Color Code **Silver**	Color Code **Silver**	Color Code **Silver**
**Bomb Threat**	Color Code **Gray**	-	Color Code **Gray**	Color Code **Black**	-
**Cardiac Arrest**	Color Code **Blue**	Color Code **Blue**	Color Code **Blue**	Color Code **Blue**	Color Code **Blue**
**Fire**	Color Code **Red**	Color Code **Red**	Color Code **Red**	Color Code **Red**	Color Code **Red**
**Hazardous Materials**	Color Code **Orange**	Color Code **Orange**	Color Code **Orange**	Color Code **Orange**	Color Code **Orange**
**Hostage Situation**	-	Color Code **Silver**	Color Code **Gray**	Color Code **Silver**	Color Code **Silver**
**Infant Abduction**	Color Code **Pink**	Color Code **Pink**	Color Code **Pink**	Color Code **Pink**	Color Code **Pink**
**Internal Disaster**	-	Code Triage B	Code Triage	Code [Facility]	Code Triage B
**Mass Casualty**	Code Triage	Code Triage A	Code Triage	Code Triage	Code Triage A
**Missing Adult**	Color Code **Gold**	Code E	Color Code **Gray**	-	-
**Missing Child**	Color Code **Gold**	Color Code **Pink**	Color Code **Pink**	Color Code **Pink**	Color Code **Pink**
**Pediatric Emergency**	-	-	-	-	-
**Severe Weather**	Color Code **Green**	Color Code **Gray**	Plain Language	Plain Language	Color Code **Gray**
**Violence**	Code Strong	Plain Language—“Security Stat”	Color Code **Gray**	Color Code **Gray**	Plain Language—“Security Stat”

**Table 4:** Results of demographic characteristics one-way ANOVA tests with code identification and change in confidence score (N = 304). Significant *p*-values (≤0.05) are bolded.

		Code Identification Score (%)			Change in Confidence Score		
	n (%)	Mean (SD) ^1^	F	*p*-Value ^2^	Mean (SD) ^1^	F	*p*-Value ^2^
**Facility**			33	**<0.001**		2	**0.094**
A	99 (33%)	32.8 (14.8)			−0.90 (1.23)		
B	21 (6.9%)	60.9 (19.8)			−0.29 (0.64)		
C	59 (19%)	58.4 (17.4)			−1.03 (1.02)		
D	78 (26%)	48.9 (15.5)			−0.77 (1.28)		
E	47 (15%)	37.2 (16.6)			−1.02 (1.11)		
**Position Type**			1	0.3		0.82	0.4
Clinical	196 (64%)	45.3 (19.4)			−0.91 (1.14)		
Non-clinical	108 (36%)	43.0 (19.0)			−0.79 (1.22)		
**Typical Shift**			2.3	0.1		0.05	>0.9
Day Shift	279 (92%)	45.1 (19.3)			−0.87 (1.14)		
Night Shift	5 (1.6%)	47.1 (17.2)			−0.80 (0.84)		
Equal times on both shifts	20 (6.6%)	35.7 (17.5)			−0.80 (1.54)		
**Facility Experience**			3	**0.031**		0.31	0.8
0–2 years	90 (30%)	40.2 (17.2)			−0.94 (1.27)		
2–5 years	77 (25%)	43.9 (19.6)			−0.86 (1.13)		
5–8 years	32 (11%)	49.6 (18.1)			−0.72 (1.08)		
>8 years	105 (35%)	47.1 (20.4)			−0.86 (1.13)		
**Healthcare Experience**			3.8	**0.011**		0.83	0.5
0–2 years	51 (17%)	40.5 (15.8)			−1.08 (1.38)		
2–5 years	67 (22%)	40.3 (16.8)			−0.85 (1.12)		
5–8 years	37 (12%)	51.4 (21.5)			−0.70 (1.05)		
>8 years	149 (49%)	46.1 (20.2)			−0.85 (1.13)		
**Total Facilities in Career**			2.4	**0.07**		1.2	0.3
1 facility	123 (40%)	48.0 (19.5)			−0.92 (1.14)		
2–3 facilities	106 (35%)	42.3 (18.1)			−0.71 (1.19)		
4–5 facilities	42 (14%)	43.0 (20.7)			−0.95 (1.21)		
>5 facilities	33 (11%)	40.5 (19.0)			−1.09 (1.07)		

^1^ Mean (Standard Deviation), ^2^ One-way ANOVA.

**Table 5:** Results of experience with emergency codes one-way ANOVA tests with code identification and change in confidence score (N = 304). Significant *p*-values (≤0.05) are bolded.

		Code Identification Score (%)			Change in Confidence Score		
	n (%)	Mean (SD) ^1^	F	*p*-Value ^2^	Mean (SD) ^1^	F	*p*-Value ^2^
**Knowledge of Code Activation Procedures**			32	**<0.001**		0.13	0.7
No	94 (31%)	35.6 (14.6)			−0.90 (1.30)		
Yes	210 (69%)	48.5 (19.8)			−0.85 (1.10)		
**Witnessed Code Confusion**			0.07	0.8		0.19	0.7
No	196 (64%)	44.3 (18.4)			−0.85 (1.10)		
Yes	108 (36%)	44.9 (20.8)			−0.91 (1.28)		
**Worked at a Facility with Different Color Codes**			2.6	0.078		1.7	0.2
No	197 (65%)	46.3 (19.2)			−0.80 (1.12)		
Yes	80 (26%)	41.2 (19.5)			−0.93 (1.27)		
Maybe	27 (8.9%)	41.0 (17.6)			−1.22 (1.09)		
**Code Type Preference**			4.7	**0.031**		0.5	0.5
Color Codes	199 (65%)	46.2 (19.4)			−0.83 (1.14)		
Plain Language	105 (35%)	41.2 (18.6)			−0.93 (1.21)		
**Code Exceptions for Plain Language**			18	**<0.001**		0.8	0.4
No	64 (21%)	35.6 (17.1)			−0.98 (1.40)		
Yes	240 (79%)	46.9 (19.1)			−0.84 (1.10)		
**Training at Orientation**			5.8	**0.017**		0.49	0.5
No	96 (32%)	40.6 (17.7)			−0.94 (1.19)		
Yes	208 (68%)	46.3 (19.7)			−0.84 (1.16)		
**Training During Drills/Exercises**			4.1	**0.044**		2.5	0.12
No	135 (44%)	42.0 (19.4)			−0.99 (1.23)		
Yes	169 (56%)	46.5 (19.0)			−0.78 (1.10)		
**Annual Training**			18	**<0.001**		4.6	**0.033**
No	149 (49%)	39.8 (17.2)			−1.01 (1.23)		
Yes	155 (51%)	49.0 (20.1)			−0.73 (1.08)		
**Time Since Last Training**			2.2	0.093		1.4	0.2
<1 month	51 (17%)	43.7 (22.9)			−0.67 (1.11)		
1–6 months	113 (37%)	47.7 (19.6)			−0.80 (1.18)		
6–12 months	83 (27%)	43.6 (17.2)			−0.94 (1.10)		
>1 year	57 (19%)	40.1 (17.2)			−1.09 (1.26)		

^1^ Mean (Standard Deviation), ^2^ One-way ANOVA.

**Table 6:** Results of regression analyses for code identification score. Significant *p*-values (≤0.05) are bolded. Only variables significantly associated with code identification score were included in the multivariate regression.

	Univariate			Multivariate		
	Beta	95% CI ^1^	*p*-Value	Beta	95% CI ^1^	*p*-Value
**Facility**						
A	—	—		—	—	
B	28.1	20.5, 35.7	**<0.001**	25.6	18.3, 33.0	**<0.001**
C	25.6	20.4, 30.8	**<0.001**	21.7	16.4, 27.0	**<0.001**
D	16.1	11.4, 20.9	**<0.001**	15.9	11.1, 20.8	**<0.001**
E	4.48	−1.12, 10.1	0.12	3.92	−1.46, 9.31	0.15
**Position Type**	−2.35	−6.87, 2.18	0.31			
**Typical Shift**						
Day Shift	—	—		—	—	
Night Shift	2.06	−14.9, 19.0	0.81	3.18	−10.4, 16.8	0.65
Equal times on both shifts	−9.37	−18.1, −0.671	**0.036**	−3.84	−11.1, 3.42	0.3
**Facility Experience**						
0–2 years	—	—		—	—	
2–5 years	3.72	−2.08, 9.52	0.21	5.85	0.028, 11.7	**0.05**
5–8 years	9.39	1.70, 17.1	**0.017**	4.09	−4.17, 12.3	0.33
>8 years	6.98	1.62, 12.4	**0.011**	2.28	−4.39, 8.95	0.5
**Healthcare Experience**						
0–2 years	—	—		—	—	
2–5 years	−0.178	−7.10, 6.74	0.96	−3.66	−10.7, 3.37	0.31
5–8 years	10.9	2.84, 18.9	**0.008**	3.72	−5.16, 12.6	0.41
> 8 years	5.59	−0.447, 11.6	0.071	0.302	−7.31, 7.91	0.94
**Total Facilities in Career**						
1 facility	—	—		—	—	
2–3 facilities	−5.65	−10.6, −0.682	**0.027**	−2.36	−6.76, 2.04	0.29
4–5 facilities	−4.94	−11.6, 1.76	0.15	−6.72	−12.9, −0.579	**0.033**
>5 facilities	−7.49	−14.8, −0.143	**0.047**	−6.29	−13.0, 0.411	0.067
**Knowledge of Code Activation Procedures**	12.8	8.37, 17.3	**<0.001**	9.07	5.01, 13.1	**<0.001**
**Witnessed Code Confusion**	0.629	−3.90, 5.16	0.79			
No	—	—		—	—	
Yes	−5.18	−10.2, −0.200	**0.042**	−1.62	−6.22, 2.98	0.49
Maybe	−5.33	−13.0, 2.37	0.18	0.726	−5.84, 7.29	0.83
**Code Type Preference**	−5.01	−9.53, −0.482	**0.031**	0.435	−3.36, 4.23	0.82
**Code Exceptions for Plain Language**	11.3	6.11, 16.4	**<0.001**	4.32	−0.246, 8.89	0.065
**Training at Orientation**	5.67	1.05, 10.3	**0.017**	4.2	0.316, 8.09	**0.035**
**Training During Drills/Exercises**	4.48	0.147, 8.82	**0.044**	2.12	−1.52, 5.76	0.26
**Annual Training**	9.15	4.94, 13.4	**<0.001**	0.781	−3.01, 4.57	0.69
**Time Since Last Training**						
<1 month	—	—				
1–6 months	4.03	−2.30, 10.4	0.21			
6–12 months	−0.066	−6.74, 6.61	0.98			
>1 year	−3.6	−10.8, 3.64	0.33			

^1^ CI = Confidence Interval.

## Data Availability

The data presented in this study are available on request from the corresponding author. The data are not publicly available due to privacy restrictions of the participating facilities.

## Supplementary Materials

The computer codes for this study can be downloaded at www.github.com/morganataylor/color-codes-public.
